# Controlled Release of Flurbiprofen from 3D-Printed and Supercritical Carbon Dioxide Processed Methacrylate-Based Polymer

**DOI:** 10.3390/pharmaceutics15041301

**Published:** 2023-04-21

**Authors:** Truc T. Ngo, Jae D. Kim

**Affiliations:** Department of Industrial and Systems Engineering, Shiley-Marcos School of Engineering, University of San Diego, 5998 Alcala Park, San Diego, CA 92110, USA; jaedkim@sandiego.edu

**Keywords:** polymer, supercritical carbon dioxide, flurbiprofen, 3D printing, controlled release, modeling

## Abstract

The ability to engineer and predict drug release behavior during treatment is critical to the design and implementation of effective drug delivery systems. In this study, a drug delivery system consisting of a methacrylate-based polymer and flurbiprofen was studied, and its release profile in a controlled phosphate-buffered saline solution was characterized. The polymer, which was 3D printed and processed in supercritical carbon dioxide under different temperature and pressure settings, showed sustained drug release over a prolonged period. A computer algorithm was used to determine the drug release time duration before reaching steady state and the maximum drug release at steady state. Several empirical models were applied to fit the release kinetic data to gain information about the drug release mechanism. The diffusion coefficients for each system were also estimated using Fick’s law. Based on the results, the influence of supercritical carbon dioxide processing conditions on the diffusion behavior is interpreted, providing insights into the effective and tunable design of drug delivery systems for targeted treatment specifications.

## 1. Introduction

The development of targeted drug delivery systems over the last two decades has helped increase the efficiency of human disease treatments, prevent post-surgery infections, and facilitate fast recovery and regeneration of new tissues. Biocompatible polymers, such as poly(methyl methacrylate) (PMMA), poly(lactic acid) (PLA), and poly(caprolactone) (PCL), have been widely used as the system carrier. They serve as the matrix to hold the drug and then release it locally inside the body under a certain condition [1].

The polymers are often prepared through a polymerization process, and are then formed into shapes such as filaments, microspheres, or films using casting, extrusion, or molding methods. These conventional methods produce solid polymer samples without the ability to control structural porosity. To address this limitation, there have been some attempts to apply 3-dimensional (3D) printing technology to prepare the polymer matrix for various biomedical, medical, and pharmaceutical applications, with more flexibility and controllability of material porosity. For example, Velu et al. used selective laser sintering to 3D-print PMMA/β-tricalcium phosphate composite for bone repair and replacement applications [2]. Valerga et al. applied a fused deposition modeling technique to fabricate a PLA base for implant devices [3].

To prevent negative effects of residual, undesirable chemicals on the human body resulting from the preparation of polymer/drug systems, supercritical carbon dioxide (scCO_2_) is used to replace typical organic solvents in the polymer processing stage. The use of scCO_2_ as a processing medium to formulate drug-impregnated polymeric materials has shown to be effective and reliable [4,5,6,7,8]. The level of drug loading can be tuned by changing scCO_2_ processing temperature, pressure, and treatment time duration [5].

Despite much effort in drug delivery system development using scCO_2_ and 3D-printed biomaterials, there has been limited work reported in literature where 3D printing is combined with scCO_2_ to produce a more flexible, tunable, and predictable drug delivery system. Schmid et al. completed a study on 3D-printed PLA-based drug release system, using scCO_2_ to incorporate ibuprofen into the polymer [9]. Fused deposition modeling, as the 3D-printing method, was employed to produce thin film, spherical and cylindrical samples with varying pore sizes. Although drug loading was not characterized in the samples, the authors reported a correlation between drug dissolution rate in phosphate buffer solution with sample pore size, using the Fickian diffusion model derived from Korsmeyer–Peppas plots [10]. In another study performed by Ngo et al., PMMA and β-tricalcium phosphate composite was 3D-printed using the selective laser sintering method [4]. The composite was then further processed in scCO_2_ to incorporate the flurbiprofen drug into the host matrix. Drug loading was characterized and found to vary linearly with drug solubility in scCO_2_ under the experimented conditions. The drug release profile was also found to follow the Weibull model reliably.

Previously, drug delivery systems were developed from a 3D-printed methacrylate-based polymer using the stereolithography method. Flurbiprofen, an anti-inflammatory drug, was then impregnated inside the polymer matrix using scCO_2_ under different processing conditions. Drug loading in the polymer was characterized and reported by Ngo et al. [5], showing correlation with scCO_2_ processing temperature, pressure, treatment time, and 3D-printing settings. Additionally, material surface roughness was found to depend on layer thickness setting used in the 3D-printing process. Linear regression analyses yielded mathematical models, predicting the level of drug loading and the material surface roughness based on 3D printing settings and scCO_2_ processing conditions.

This current study focuses on the characterization of the drug release profile for the previously developed polymer/flurbiprofen drug delivery systems under varying scCO_2_ processing temperatures and pressures. The novelty of this work is the comprehensive approach to drug release modeling and estimations of diffusion coefficients for varied drug delivery systems. Past studies on other delivery systems used either empirical methods [11,12] or analytical methods [13,14,15,16,17] to model release data, but not a combination of both. In this study, a dynamic computer algorithm was first employed to determine the drug release time before each system reached steady state and the maximum level of drug release at steady state. Several empirical models were then used to fit the release profile for each system to determine the most probable drug release mechanism. Diffusion coefficients were also calculated for each system using Fick’s law and diffusion-based modeling. The goal of this work is to derive correlations between scCO_2_ processing conditions during the preparation stage of the drug delivery systems and the release behavior during the active release stage. The calculated diffusion parameters can be used to predict the release time for a known release target, or the level of drug release for a targeted treatment duration. As a result, drug delivery systems can be engineered accordingly to meet specifications of various applications.

## 2. Materials and Methods

### 2.1. Materials

Polymer samples were printed on a FormLabs Form 2 3D printer, using stereolithography. Details about the 3D printing process can be found in another study previously published by Ngo et al. [5]. The starting resin, Clear Resin v4, contained a mixture of monomers and oligomers of methacrylic acid esters, along with a photoinitiator to facilitate the polymerization process under ultraviolet light activation. Materials were prepared with a 0.100-mm printing layer thickness setting. Printed samples had length × width dimensions of 20 mm × 10 mm, which were subsequently divided into equal halves of 10 mm × 10 mm before processing. Samples’ average thickness was measured to be 0.274 ± 0.009 mm and 0.333 ± 0.013 mm in two different printing batches [5]. Sample thickness variability from batch to batch was a limitation of the FormLabs Form 2 3D printer, despite identical printer settings and printing conditions being employed in all batches. However, within-sample and sample-to-sample thickness uniformity within each batch was found to be excellent, with variation under 4%.

Flurbiprofen (purchased from Sigma-Aldrich, Saint Louis, MO, USA) was impregnated into the polymer matrix using the scCO_2_ processing method described in Ngo et al. [5]. ScCO_2_ processing was performed at two different temperature settings, 313 K and 323 K, coupled with four distinct pressure conditions: 115 bar, 125 bar, 135 bar, and 148 bar. Samples were treated under constant temperature and static pressure for 24 h to ensure drug loading saturation. At the end of the treatment, drug-impregnated polymer samples were removed from the scCO_2_ reactor chamber. A gentle blow of compressed air was applied to remove loose drug powders at the surface. Drug loading amount was calculated for different sample types under varying scCO_2_ treatment conditions. Complete results were reported by Ngo et al. [5], with the data summary shown in Table 1.

### 2.2. Drug Release Measurements

The flurbiprofen-impregnated polymer samples were soaked in 80 mL of phosphate-buffered saline (PBS, pH 7.2, purchased from ThermoFisher Scientific, Carlsbad, CA, USA) for up to 45 days. PBS solution was maintained at 310 ± 1 K on a hot plate with constant stirring using a magnetic bar, rotating at 150 revolutions per minute. As flurbiprofen dissolved into PBS, solution samples were drawn, and their ultraviolet (UV) absorbance was measured using a USB 2000 + Ocean Optics UV-visible spectrometer with UV-transparent optical fibers. Three one-ml samples were taken for each measurement and the solution was replenished with pure PBS to compensate for the amount withdrawn. Two to three measurements were performed during the first 24 h of the sample soaking period. After that, one measurement was taken for each one- to three-day time interval until drug release appeared to have stabilized.

To determine the amount of flurbiprofen present in each PBS solution sample, a calibration curve for flurbiprofen in PBS was established with three repeated measurements for each data point. Flurbiprofen shows a strong UV absorption peak at 250 nm. Its UV absorbance is directly proportional to its concentration in PBS, following Beer-Lambert law, as shown in Equation (1).

Flurbiprofen’s UV absorbance @ 250 nm = 25.736 × flurbiprofen concentration(1)

No change in flurbiprofen’s UV absorption peak wavelength or shape was observed throughout the drug release stage in comparison with its original drug form before scCO_2_ processing. The cumulative amount of flurbiprofen released into PBS solution (i.e., mass of released drug) by the sampling time was derived from the concentration value obtained in Equation (1). At the higher concentration range, sampling solutions were diluted accordingly to lower the UV absorbance to an unsaturated level for more reliable quantification. UV absorbances were averaged over 24 scans and a one-second integration time. The relative fraction of drug release as a function of time was calculated using Equation (2):

(2) $$\frac{M_{t}}{M_{o}}=\frac{\mathrm{mass}\;\mathrm{of}\;\mathrm{released}\;\mathrm{drug}}{\mathrm{pre}-\mathrm{treatment}\;\mathrm{mass}\;\mathrm{of}\;\mathrm{polymer}\;\times \;\mathrm{drug}\;\mathrm{loading}}$$

where *M_t_* represents the cumulative mass of the drug released into the solution by time *t*, and *M_o_* represents the total mass of the drug present inside the polymer sample at time zero.

Because the actual drug loading was not possibly determined for the exact same sample that the drug release study was performed on, the initial drug loading, *M_o_*, used in Equation (2) was taken from its “twin” sample, which underwent identical 3D printing and scCO_2_ treatment processes within the same batch. The method used to determine drug loading was previously reported by Ngo et al. [5].

Eight different types of polymer/flurbiprofen samples were used in the drug release study, differentiated by scCO_2_ processing temperatures and pressures. Sample attributes are summarized in Table 1 with letter labels for convenient references. CO_2_ density values shown in Table 1 were either obtained directly or interpolated from Anwar and Carroll reference data [18]. Here, the drug loading value represents the ratio between the mass of the drug loaded inside the polymer sample and the original, untreated polymer mass, expressed as a percentage. Three repeated runs were performed for each sample type, with an exception for sample type A, where data were collected over two repeated experiments only. 3D printing conditions were kept the same for all material systems.

### 2.3. Steady State Determination

All release data was first normalized to a maximum release of 100% for consistent data processing. Experimental data was analyzed to determine the transitional time between the initial drug release and steady state, *T_ss_*, and the total amount of drug release when the diffusion process entered steady state, *M_ss_*. The determination of *T_ss_* and *M_ss_* required a segmentation of the release process into two distinct phases. In the first phase, the drug release commenced with an initial burst with a declining slope, exhibiting a logarithmic trend. In the later phase, the release process entered a “steady state” where the change in rate of release showed no statistical significance. For each sample’s experimental dataset, the following Algorithm 1 was applied to calculate *T_ss_* and *M_ss_*:
**Algorithm 1: Pseudo-code to determine sample’s steady-state region**For each sample n:Sort the drug released value based on time (t) in ascending order.Assign the first two initial data points, starting from time zero (t = 0), into set A which corresponds to region A.Assign the remaining data points to set B which corresponds to region B.While the *p*-value of region B > significance level (0.05):
○Remove the first data point from set B and assign to set A.○Create a linear regression model using the data in set A (model A).○Create a linear regression model using the data in set B (model B).○If the *p*-value of model B is above the significance level (0.05), move to the next iteration.○If the *p*-value of model B is below the significance level (0.05), terminate the while loop.○If all data points have been assigned to set A, terminate the while loop because there is no more data in region B.
Move to the next sample.End

**Figure 1:**
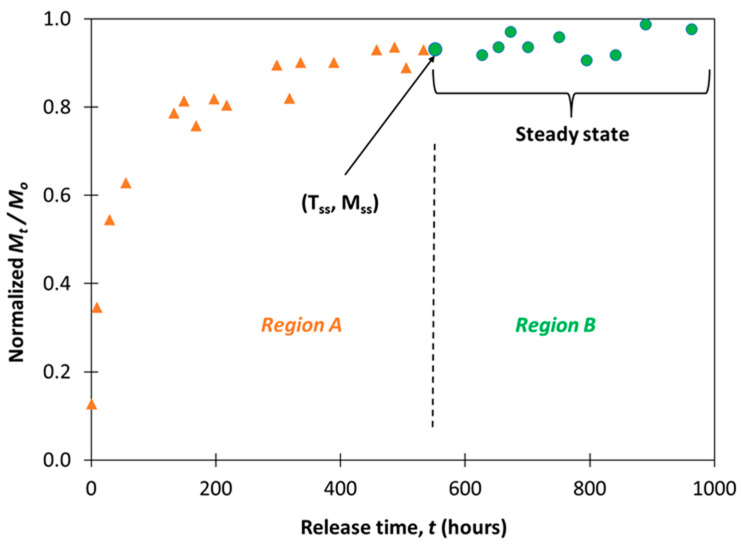
Example of algorithm execution outcome for steady state determination.

The algorithm essentially checks whether the least square line for the data points in set B has a non-zero slope. If the slope is zero, drug release data in set B is no longer dependent on time. Therefore, we can conclude that the corresponding region B is set to “steady state”. Figure 1 illustrates the algorithm outcome for one sample’s experimental dataset. *T_ss_* and *M_ss_* correspond to the x- and y-values of the first data point in the “steady state” region (i.e., region B).

### 2.4. Modeling of Drug Release

#### 2.4.1. Empirical Modeling

Drug release kinetic data for each sample was fitted to five empirical models: zero-order, first-order, Higuchi, Korsmeyer-Peppas, and Weibull [19]. These models were selected based on the type of polymer samples used in the study and the slow release of the drug from the polymer.

##### Zero-Order

The drug release process can be modeled using a simple linear equation:

(3) $$\frac{M_{t}}{M_{o}}=kt$$

where *k* is the zero-order constant. The zero-order model has been used to describe drug release from several types of modified pharmaceutical dosage forms, such as transdermal systems, matrix tablets of soluble drugs, coated forms, and osmotic systems [20].

##### First-Order

The first-order model assumes a constant release rate and can be expressed as a logarithmic function:

(4) $$\log C_{t}=\log C_{0}-\frac{kt}{2.303}$$

where $C_{t}$ is the drug concentration remaining in the matrix at time t, $C_{0}$ is the initial drug concentration, and k is the first-order constant. The model has been used to describe drug dissolution from porous matrices [19].

##### Higuchi

The drug release process via diffusion in one dimension can be modeled using a method proposed by Higuchi:

(5) $$Q_{t}=kt^{1/2}$$

where $Q_{t}$ represents the fraction of total drug released by time t and k is the Higuchi dissolution constant. The model has been used to describe the drug release from pharmaceutical dosage forms, such as transdermal systems and matrix tablets with water soluble drugs [19].

##### Korsmeyer-Peppas

In the Korsmeyer-Peppas model [10], the first 60% of the drug release data is used to fit a simple exponential equation with a varying release exponent value:

(6) $$\frac{M_{t}}{M_{o}}=kt^{n}$$

where k is a rate constant and n is the release exponent. If n<0.45, the release corresponds to Fickian diffusion. If 0.45<n<0.89, the release corresponds to non-Fickian anomalous transport. If n=0.89, the release is Case II transport. If n>0.89, then the release is super Case II transport.

##### Weibull

The Weibull model has been used to compare the release profiles of matrix type drug delivery system [19]. The general Weibull relationship is expressed as follows:

(7) $$M_{t}=M_{o}\left[1-e^{-\frac{{\left(t-T\right)}^{b}}{a}}\right]$$

where T is the time lag (which is zero in this study), a is the scale parameter, and b is the shape parameter.

#### 2.4.2. Diffusion-Based Modeling

Another modeling approach taken in this study was based on Fick’s second law of diffusion. Drug release was divided into two stages before reaching steady state: stage I for 0 ≤ *M_t_*/*M_o_* ≤ 0.6 and stage II for 0.6 < *M_t_*/*M_o_* ≤ steady state. For each of the release stages, the diffusion coefficients were calculated based on Equation (8) (stage I) and Equation (9) (stage II) [21]:

(8) $$\frac{M_{t}}{M_{o}}=4{\left(\frac{D_{I}t}{\pi h^{2}}\right)}^{1/2}$$

(9) $$\frac{M_{t}}{M_{o}}=1-\frac{8}{\pi^{2}}\;exp\left(\frac{-\pi^{2}D_{II\;}t}{h^{2}}\right)$$

In these equations, *D_I_* and *D_II_* represent the diffusion coefficient of the drug being released from the polymer matrix into the solution during stage I and stage II, respectively, and *h* is the polymer sample thickness. Rearranging Equation (8) to obtain a linear time relationship, we see:

(10) $${\left(\frac{M_{t}}{M_{o}}\right)}^{2}=\frac{16D_{I}}{\pi h^{2}}\cdot t$$

**Figure 2:**
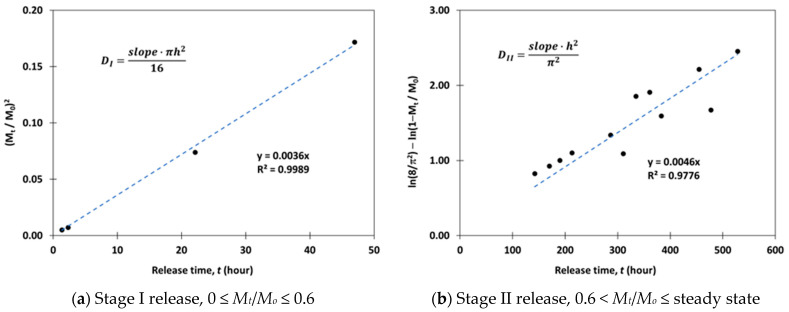
Examples of diffusion coefficient estimations using Fick’s law and drug release data.

The kinetic drug release data for 0 ≤ *M_t_/M_o_* ≤ 0.6 was plotted as a function of time. An example is shown in Figure 2a. The diffusion coefficient *D_I_* for stage I release was then derived from the slope of the best fit linear trendline. A similar analytical method was applied to determine the diffusion coefficient *D_II_* for stage II release. In this case, Equation (9) is rearranged to form a linear time relationship, as shown in Equation (11). *D_II_* value was calculated from the slope of the best fit linear trendline for the drug release dataset within the range of 0.6 < *M_t_*/*M_o_* ≤ steady state. An example is shown in Figure 2b.

(11) $$\ln\frac{8}{\pi^{2}}-\ln\left(1-\frac{M_{t}}{M_{o}}\right)=\frac{\pi^{2}D_{II}}{h^{2}}\cdot t$$

To further improve the overall accuracy of the diffusion model, a numerical method was applied to fit the drug release kinetic data for each sample, using *D_I_* and *D_II_* values calculated analytically as the initial estimates. The numerical search process was performed separately for *D_I_* and *D_II_*. Starting with the initial values of *D_I_* and *D_II_*, Equations (8) and (9) were used, respectively, to calculate the predicted relative drug release fraction $\left(M_{t}/M_{o}\right)$ for each known t from the drug release data for each sample. The total sum of squared errors (SSE) was then calculated:

(12) $$SSE=\sum {\left(y-\hat{y}\right)}^{2}$$

where y represents the actual $\left(M_{t}/M_{o}\right)$ and $\hat{y}$ represents the predicted $\left(M_{t}/M_{o}\right)$ at a known time t. A small step increment $\left(\alpha =10^{-6}\right)$ was used to change the value of *D_I_* and *D_II_* in both directions $\left(\pm \alpha \right)$ and the corresponding SSE was recalculated. If there was an improvement in the SSE value, the search continued until there was no longer an improvement in any direction. The resulting coefficient of determination (R^2^) was also determined for the final D_I_ and D_II_ estimations.

## 3. Results and Discussion

### 3.1. Drug Release Time and Dosage at Steady State

**Figure 3:**
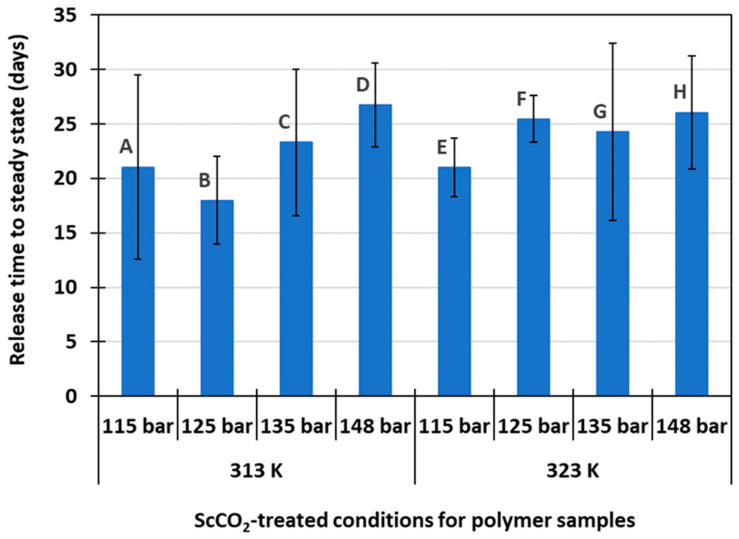
Release time to steady state for samples treated under different scCO_2_ conditions.

On average, it took approximately 24 days for the drug release process to reach steady state. Figure 3 shows the time duration of drug release for each of the eight drug delivery systems. A high variability within each run was due to random variations among the individual human samplers over a prolonged period of experimental time. Original polymer sample thickness did not appear to play a role in the variation of release times within each run. Despite data variability, polymer samples that were previously treated in scCO_2_ at a lower pressure (115–125 bar at 313 K and 115 bar at 323 K) appear to reach steady state sooner than those treated at higher pressures. The initial amount of drug present in the polymer host, as seen in Table 1, does not seem to correlate with the shorter release time. This phenomenon could be explained through the swelling behavior of the polymer matrix during scCO_2_ treatment. The swelling behavior of PMMA in scCO_2_ has been well studied and characterized [22,23,24,25,26]. Both temperature and pressure have influential effects on the swelling of the polymer matrix. Shinkai et al. shows that the PMMA swelling ratio, defined as the percentage change in sample thickness, increases with increasing pressure up to 300 bar [22]. In another study conducted by López-Periago et al., PMMA swelling occurs non-homogeneously across the polymer thickness [27]. The swelling starts from the outside peripheral and progresses inward. Applying these phenomena to the host polymer matrix used in this study, at lower pressure conditions, drug sorption into the polymer matrix during scCO_2_ treatment likely occurred closer to the material surface compared to those treated at higher pressures where more swelling would occur. Due to the shallower penetration of the drug molecules in the polymer matrix, they were released quicker into PBS solution.

**Figure 4:**
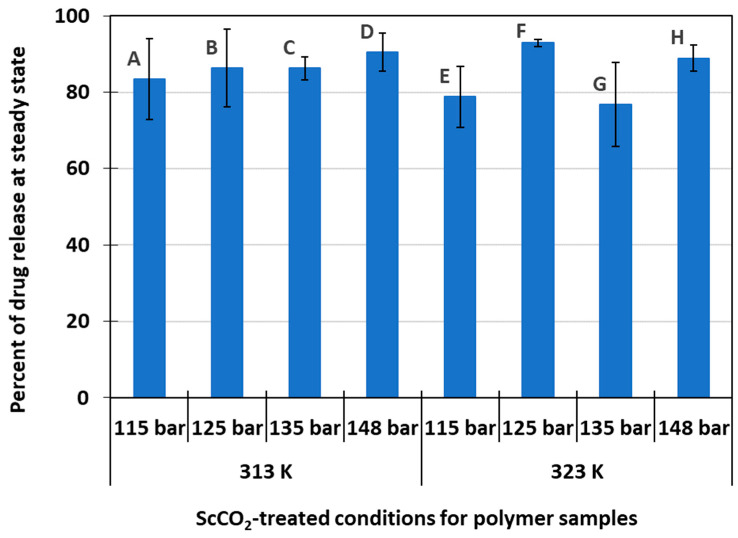
Steady state drug release level for samples treated under different scCO_2_ conditions.

Figure 4 shows the average percent of drug release at steady state for each of the eight delivery systems experimented with in this study. Overall, data shows that these systems were able to release 85.5 ± 5.6% of their original drug amount present inside the polymer matrix. A high level of drug release capability over a sustained period of time offers great potential for efficient drug delivery systems where most of the loaded drug dosages are released, interact with the target site, and perform their intended function. Release efficiency for delivery systems A, B, C, D was slightly higher than that for systems E, F, G, H (86.6% versus 84.3%). However, the difference was not statistically significant to claim that scCO_2_-processing temperature played a significant role in drug release efficiency (*p*-value > 0.05). Polymer swelling effect of CO_2_ on the host polymer matrix during scCO_2_ treatment could also be the cause of the observed small difference in release efficiency. Li et al. shows that the volume change ratio of PMMA due to swelling in CO_2_ increases with temperatures between 310 K and 370 K [26]. A lower processing temperature during scCO_2_ treatment would induce less swelling of the polymer matrix. Since polymer swelling in CO_2_ tends to start at the exposed surfaces of the sample before extending inward [27], one can assume that the swelling of the polymer host matrix at a lower scCO_2_ processing temperature resulted in a shallower drug absorption. Therefore, the drug molecules in these samples were able to diffuse more easily into PBS solution during the release stage. On the other hand, the drug molecules were incorporated deeper inside the polymer matrix at a higher temperature during scCO_2_ treatment, making them harder to diffuse out of the matrix during release. As a result, systems A, B, C, D showed a greater drug release efficiency compared to systems E, F, G, H.

Although the drug release level at steady state was comparable for systems A–D, some slight variations were observed among systems E–H. Specifically, system F had a slightly higher drug release level at steady state compared to the other systems processed at the same 323 K temperature setting. This behavior could be attributed to the combined effect of relatively higher drug loading in the initial sample and lower pressure condition. As explained earlier, lower pressure would induce less swelling of the host polymer matrix [22], causing the drug molecules to reside at a shallower depth inside the material surface post scCO_2_ treatment stage. As a result, these drug molecules became dissolved in PBS solution and released from the host polymer matrix more efficiently. Moreover, a higher initial drug loading means a higher amount of drug available to be released from the system upon dissolution in PBS. Although system G also had a relatively higher initial drug loading, higher-pressure treatment in scCO_2_ could have induced more in-depth swelling of the polymer matrix, resulting in deeper penetration of the drug molecules. Therefore, drug release might not have been as efficient in system G due to more hindered diffusion paths. The differences in release behavior were less apparent for systems A–D, likely due to its lower scCO_2_ processing temperature setting, which means less swelling of the polymer matrix overall.

### 3.2. Empirical Modeling of Drug Release

Drug release kinetic data for each sample in this study was fitted to five empirical models: zero-order, first-order, Higuchi, Korsmeyer-Peppas, and Weibull. Zero-order model did not appear to be a valid fit due to mostly negative R^2^ values. This result indicates that drug release was not simply due to dissolution from dosage forms [19,20]. It could also imply that the drug molecules did not simply reside at the surface of the polymer but were rather impregnated inside the polymer matrix, confirming the conclusion previously drawn by Ngo et al. [5]. Table 2 shows a summary of kinetic parameters of flurbiprofen release from the 3D printed methacrylate-based polymer, obtained from four of the five tested empirical models. Average values for the model parameters are presented in Table 2. Since data modeling for individual samples within each dataset did not yield the same R^2^ value, an R^2^ range is included for each of the fitted parameter sets. Data for the zero-order model is not shown due to its invalid fitting outcome.

Higuchi and Korsmeyer-Peppas models did not produce good fits for the drug release kinetics, with some R^2^ values in the negative range. However, first-order and Weibull models yielded the best fits, with R^2^ values mostly above 0.90. Release kinetic data agreed well with the first-order model, confirming the porous nature of the host polymer matrix produced by 3D printing. The shape parameter, b, derived from the Weibull model, was found to be between 0.47 and 0.57. With a shape parameter of less than 0.75, the kinetic modeling outcome suggests a Fickian diffusion for the release mechanism of flurbiprofen from the 3D printed methacrylate-based polymer matrix [12]. Considering the scale parameter, a, of the Weibull model, although the values pertaining to systems prepared at the lower temperature (A, B, C, D) were slightly higher than those prepared at the higher temperature (E, F, G, H), by approximately 8%, the difference is not statistically significant (*p*-value > 0.05). There was also no statistically significant difference based on scCO_2_ pressure used during material preparation. Since the scale parameter strongly depends on the surface of the host polymer matrix [28], the observations from Weibull modeling indicate that there was no significant difference among the polymer sample surfaces in this study.

### 3.3. Diffusion-Based Modeling of Drug Release

Diffusion coefficients for both stage I and stage II release prior to steady state were calculated using a combination of analytical and numerical methods based on Fick’s law for improved accuracy. Table 3 shows a summary of diffusion coefficient estimations.

Results show that diffusion rate of flurbiprofen from the polymer matrix into PBS reduced significantly between stage I and stage II, except for system D. System D was prepared under the highest scCO_2_ density condition compared to the remaining systems. D also had the lowest flurbiprofen loading prior to release and released at a slowest rate compared to the others. The unchanged drug diffusion rate between stage I and stage II could indicate a strong molecular interaction between flurbiprofen molecules and the polymer matrix. A high-density scCO_2_ processing condition might have helped keep flurbiprofen molecules locked inside the pores of the 3D-printed polymer. As a result, flurbiprofen released at a slower rate and sustained that rate throughout the process. This observation suggests that 3D-printed drug delivery systems prepared at a higher CO_2_ density condition could yield a constant and steady release rate over a prolonged period.

Stage I release lasted 2 to10 days depending on delivery system types, whereas stage II release took as long as 10 to 30 days before reaching steady state. For most of the systems, the drug release profile showed an initial faster rate, followed by a slower rate. Because drug release is a diffusion process, it is mainly driven by the drug concentration differential driving force between the host polymer phase and the PBS phase. Initially, all the drug molecules resided inside the polymer phase, resulting in the largest driving force and, thus, the largest diffusion rate. As time progressed, drug concentration in the PBS phase increased, whereas the drug concentration inside the host polymer phase decreased. Consequently, the diffusion slowed down until the system reached equilibrium where there was an equal amount of drug molecules transported in and out of the two phases.

Similar drug release behavior has been observed and reported in the literature [6,7,13,14,15,16,17,29,30,31]. However, time duration of the drug release for each stage and the magnitude of the reduction in the drug diffusion rate from stage I to stage II varied greatly, depending on the host matrix and the drug itself. For example, Rao et al. demonstrated a relatively fast release of 5-fluorouracil, an anticancer drug, from PMMA-based microgels within 18 h [16]. In this case, drug loading was through an adsorption process. On the other hand, Bouledjouidja et al. observed up to 40 days of release for dexamethasone 21-phosphate disodium, an anti-inflammatory drug, and ciprofloxacin, an antibiotic drug, from PMMA [7]. In this scenario, the drugs were impregnated into PMMA via scCO_2_, in much the same way that flurbiprofen was loaded into the methacrylate-based polymer used in the current study. The magnitude of drug release was much lower in Bouledjouidja et al.’s study, most likely because of the different polymer host preparation method and drug impregnation conditions. However, the similarly observed drug release behavior over a prolonged period confirmed the effectiveness of drug impregnation via scCO_2_. The enhanced drug loading and controlled release seen in this current study is most likely due to the 3D-printed nature of the polymer matrix. 3D printing generates more uniform pores inside the matrix, allowing a more stabilized entrapment of drug molecules inside the host.

ScCO_2_ processing temperature appeared to have some influence on the diffusion coefficient of stage I (*D_I_*) but not of stage II (*D_II_*). Specifically, *D_I_* for drug delivery systems prepared at scCO_2_ temperature of 323 K was 29% higher, on average, than those prepared at 313 K. As shown in Table 1, systems prepared at 323 K (E, F, G, H) had a higher level of flurbiprofen loading compared to systems prepared at 313 K (A, B, C, D). The initial higher drug load inside the host polymer matrix created a larger driving force for flurbiprofen to diffuse from the polymer phase to the PBS phase, resulting in a higher *D_I_*. After the initial faster release stage, the drug concentration differential between the polymer and the PBS phases became similar for all systems. Therefore, a similar *D_II_* value was observed for all system types.

The influence of scCO_2_ processing pressure on the diffusion coefficients of drug release was less significant. However, it was generally observed that systems prepared under higher scCO_2_ pressure had a slower diffusion rate during the initial stage I release. This pressure influence is consistent with the results seen with steady state achievement. A slower drug diffusion rate resulted in a longer time before reaching the steady state. These observations provide insights into the controlled drug delivery system design for targeted applications. When slower, sustained release is desired over a longer period, higher pressure condition should be used during the scCO_2_ drug impregnation of the host polymer matrix. However, other factors, such as temperature and drug solubility in scCO_2_, should also be considered as they could influence the drug partitioning effect during release, as shown in this study and prior literature reports [4,5].

## 4. Conclusions

The release kinetics of flurbiprofen from 3D-printed and scCO_2_-processed methacrylate-based polymer was studied, characterized, and modeled using a combination of empirical, analytical, and diffusion-based methods. Flurbiprofen exhibited controlled release behavior over a prolonged period of time. Drug release sustained over 24 days on average before reaching steady state, releasing more than 85% of the initially loaded drug amount. Release kinetics fit well to the first-order and the Weibull models, showing Fickian diffusion behavior and no significant influence of scCO_2_ processing conditions on the porous polymer surfaces.

Diffusion coefficients were estimated for different drug delivery systems using a combined two-stage analytical method and numerical method, based on Fick’s second law of diffusion. A faster release was observed within the initial 2–10 days, accomplishing 60% of drug release, followed by a slower rate for up to 30 days before reaching steady state. Higher scCO_2_ processing temperature and lower pressure resulted in a faster diffusion rate during the initial release stage. Similarly, higher levels of initial drug loading inside the host polymer matrix also seemed to result in a higher diffusion rate. However, scCO_2_ processing conditions did not appear to influence the drug release rate after the initial burst.

The findings from this study provide useful insights into the design of effective drug delivery systems. Different applications may require different drug release profiles, such as time duration of the initial burst, time to reach steady state, and dosage target. The calculated diffusion coefficients can be used as initial estimates to determine the expected release dosage of the drug if the treatment duration is known, or to figure out the active treatment duration if a drug dosage is set. Drug dosage can further be engineered through the 3D printing process optimization, manipulating the size and level of porosity inside the host delivery matrix.

## Figures and Tables

**Table 1:** Sample parameters used in drug release study.

Drug Delivery System	ScCO_2_ Processing Temperature (K)	ScCO_2_ Processing Pressure (bar)	CO_2_ Density (kg/m^3^)	Average Drug Loading × 100 (%)
A	313	115	702.2	16.00 ± 0.92
B	313	125	731.2	16.67 ± 1.05
C	313	135	753.6	15.63 ± 3.05
D	313	148	777.0	14.18 ± 0.64
E	323	115	548.8	21.02 ± 2.35
F	323	125	613.0	23.28 ± 0.61
G	323	135	655.5	24.08 ± 1.43
H	323	148	694.6	21.75 ± 4.07

**Table 2:** Summary of kinetic parameters for flurbiprofen release from polymer samples.

Drug Delivery System	First-Order	Higuchi	Korsmeyer-Peppas	Weibull
A	k = (1.02 ± 0.24) × 10^−2^ R^2^ = [0.88, 0.96]	k = (4.28 ± 0.55) × 10^−2^ R^2^ = [0.34, 0.87]	k = 0.52 ± 0.11 n = 1.07 ± 0.03 R^2^ = [0.17, 0.39]	a = 147.91 ± 2.91 b = 0.48 ± 0.07 R^2^ = 0.91
B	k = (1.25 ± 0.30) × 10^−2^ R^2^ = [0.92, 0.97]	k = (4.59 ± 0.36) × 10^−2^ R^2^ = [0.80, 0.90]	k = 0.56 ± 0.11 n = 1.05 ± 0.04 R^2^ = [0.08, 0.40]	a = 132.94 ± 39.63 b = 0.56 ± 0.05 R^2^ = [0.95, 0.98]
C	k = (8.85 ± 3.20) × 10^−3^ R^2^ = [0.92, 0.93]	k = (3.92 ± 0.81) × 10^−2^ R^2^ = [0.19, 0.88]	k = 0.58 ± 0.13 n = 1.04 ± 0.02 R^2^ = [0.08, 0.28]	a = 199.73 ± 129.77 b = 0.49 ± 0.07 R^2^ = [0.92, 0.97]
D	k = (8.75 ± 1.30) × 10^−3^ R^2^ = [0.92, 0.98]	k = (3.91 ± 0.26) × 10^−2^ R^2^ = [0.47, 0.90]	k = 0.53 ± 0.03 n = 1.05 ± 0.00 R^2^ = [0.26, 0.29]	a = 199.16 ± 40.41 b = 0.57 ± 0.10 R^2^ = [0.80, 0.97]
E	k = (1.04 ± 0.40) × 10^−2^ R^2^ = [0.87, 0.94]	k = (4.24 ± 0.66) × 10^−2^ R^2^ = [0.46, 0.72]	k = 0.79 ± 0.60 n = 0.98 ± 0.18 R^2^ = [−5.05, 0.44]	a = 171.49 ± 62.81 b = 0.49 ± 0.10 R^2^ = [0.86, 0.95]
F	k = (1.16 ± 0.18) × 10^−2^ R^2^ = [0.93, 0.96]	k = (4.45 ± 0.34) × 10^−2^ R^2^ = [−0.20, 0.66]	k = 0.72 ± 0.06 n = 1.02 ± 0.02 R^2^ = [0.08, 0.18]	a = 90.18 ± 43.07 b = 0.47 ± 0.04 R^2^ = [0.97, 0.98]
G	k = (7.92 ± 1.67) × 10^−3^ R^2^ = [0.91, 0.94]	k = (3.84 ± 0.42) × 10^−2^ R^2^ = [0.39, 0.88]	k = 0.44 ± 0.23 n = 1.09 ± 0.07 R^2^ = [0.09, 0.61]	a = 225.27 ± 108.59 b = 0.48 ± 0.06 R^2^ = [0.96, 0.99]
H	k = (1.01 ± 0.10) × 10^−2^ R^2^ = [0.95, 0.96]	k = (4.20 ± 0.27) × 10^−2^ R^2^ = [0.70, 0.79]	k = 0.57 ± 0.08 n = 1.06 ± 0.02 R^2^ = [0.17, 0.41]	a = 141.46 ± 13.68 b = 0.56 ± 0.01 R^2^ = [0.98, 0.99]

**Table 3:** Estimations of diffusion coefficients using a combination of analytical and numerical methods.

Drug Delivery System	*D_I_* (cm^2^/s), for Stage I Release(0 ≤ *M_t_*/*M_o_* ≤ 0.6)	*D_II_* (cm^2^/s), for Stage II Release (0.6 < *M_t_*/*M_o_* ≤ Steady State)	Diffusion Rate Reduction from Stage I to Stage II
A	(1.82 ± 0.14) × 10^−10^	(1.29 ± 0.16) × 10^−10^	29.2%
B	(2.07 ± 0.37) × 10^−10^	(1.35 ± 0.16) × 10^−10^	34.6%
C	(1.86 ± 0.94) × 10^−10^	(1.01 ± 0.36) × 10^−10^	45.6%
D	(1.10 ± 0.29) × 10^−10^	(1.10 ± 0.10) × 10^−10^	0%
E	(2.09 ± 1.51) × 10^−10^	(1.33 ± 0.57) × 10^−10^	36.6%
F	(3.33 ± 0.97) × 10^−10^	(1.44 ± 0.18) × 10^−10^	56.7%
G	(1.34 ± 0.96) × 10^−10^	(6.54 ± 2.03) × 10^−11^	51.1%
H	(2.04 ± 0.22) × 10^−10^	(1.39 ± 0.12) × 10^−10^	32.2%

## Data Availability

All data generated in this study can be found inside the manuscript.
